# Improving robustness against electrode shift of high density EMG for myoelectric control through common spatial patterns

**DOI:** 10.1186/s12984-015-0102-9

**Published:** 2015-12-02

**Authors:** Lizhi Pan, Dingguo Zhang, Ning Jiang, Xinjun Sheng, Xiangyang Zhu

**Affiliations:** State Key Laboratory of Mechanical System and Vibration, School of Mechanical Engineering, Shanghai Jiao Tong University, Shanghai, 200240 China; Department of Systems Design Engineering, Center for Bioengineering & Biotechnology, University of Waterloo, Waterloo, Canada

**Keywords:** Electromyography (EMG), Common spatial patterns (CSP), Electrode shift, Pattern recognition, Myoelectric control

## Abstract

**Background:**

Most prosthetic myoelectric control studies have concentrated on low density (less than 16 electrodes, LD) electromyography (EMG) signals, due to its better clinical applicability and low computation complexity compared with high density (more than 16 electrodes, HD) EMG signals. Since HD EMG electrodes have been developed more conveniently to wear with respect to the previous versions recently, HD EMG signals become an alternative for myoelectric prostheses. The electrode shift, which may occur during repositioning or donning/doffing of the prosthetic socket, is one of the main reasons for degradation in classification accuracy (CA).

**Methods:**

HD EMG signals acquired from the forearm of the subjects were used for pattern recognition-based myoelectric control in this study. Multiclass common spatial patterns (CSP) with two types of schemes, namely one versus one (CSP-OvO) and one versus rest (CSP-OvR), were used for feature extraction to improve the robustness against electrode shift for myoelectric control. Shift transversal (ST1 and ST2) and longitudinal (SL1 and SL2) to the direction of the muscle fibers were taken into consideration. We tested nine intact-limb subjects for eleven hand and wrist motions. The CSP features (CSP-OvO and CSP-OvR) were compared with three commonly used features, namely time-domain (TD) features, time-domain autoregressive (TDAR) features and variogram (Variog) features.

**Results:**

Compared with the TD features, the CSP features significantly improved the CA over 10 % in all shift configurations (ST1, ST2, SL1 and SL2). Compared with the TDAR features, a. the CSP-OvO feature significantly improved the average CA over 5 % in all shift configurations; b. the CSP-OvR feature significantly improved the average CA in shift configurations ST1, SL1 and SL2. Compared with the Variog features, the CSP features significantly improved the average CA in longitudinal shift configurations (SL1 and SL2).

**Conclusion:**

The results demonstrated that the CSP features significantly improved the robustness against electrode shift for myoelectric control with respect to the commonly used features.

## Introduction

Surface electromyography (EMG) signals, which contain neural information [1], have long been used as control inputs of myoelectric prostheses [2–4]. With most conventional, commercially available myoelectric prostheses, a control scheme based on using amplitude or power of the EMG signals to control one degree-of-freedom (DOF) has been employed for several decades. To improve the functionality and provide more intuitive control of myoelectric prostheses, pattern recognition methods have been employed to classify EMG signals towards multifunctional prosthesis control for more than 20 years [5–9]. The pattern recognition-based control scheme is based on the assumption that amputees can activate consistent (same motion) and distinctive (different motions) EMG patterns using residual stump muscles [10].

In general, there are two types of surface EMG, low density (less than 16 electrodes, LD) EMG and high density (more than 16 electrodes, HD) EMG, which are classified by the number of electrodes. Electrode shift is an identified problem existing in both LD and HD EMG applications. It may occur during repositioning or donning/doffing of the prosthetic socket. It is one of the main reasons for degradation in classification accuracy (CA) [11]. In LD EMG, some researchers proposed efficient methods to reduce the CA degradation of electrode shift. Hargrove et al. proposed a strategy training the classifier with EMG signals from all expected displacement locations [12]. However, this strategy needing long-time training can be often frustrating for the user and leading to frequent device abandonment [13]. Young et al. demonstrated that electrode with larger size reduced the sensitivity of shift while performing worse than electrodes with smaller size without shift [11]. They suggested that electrodes oriented in longitudinal direction with the muscle fibers performed better than that oriented in transversal direction. They also showed that time-domain autoregressive (TDAR) features achieved the best classification performance and was least affected by electrode displacements. They further demonstrated that a greater interelectrode distance improved classification performance, and a combination of longitudinal and transversal electrode configurations also improved the performance in the presence of electrode shift [7].

Recently, HD EMG signals become an alternative for myoelectric prostheses [14–19]. Huang et al. showed that double differential spatial filter on HD EMG signals could improve the myoelectric control performance on targeted muscle reinnervation (TMR) patients [14]. However, for HD EMG application, the electrode shift is also very common and serious. Stango et al. used variogram (Variog) of HD EMG signals to provide features robust to electrode number and shift for myoelectric control [18]. The Variog is a statistical measure of the spatial correlation and widely used as spatial-domain feature for classification in geostatistic [20, 21]. It can be also called semivariance since it is a graph of the semivariance against the distance.

To solve the electrode shift problem of HD EMG, common spatial patterns (CSP), a method widely used in electroencephalogram (EEG) study has drawn our attention [22, 23]. In general, EEG has many electrodes (64 ∼ 128), which are similar to the HD EMG condition. Therefore, we expect the excellent capacity of CSP in EEG can also be suitable for HD EMG. Actually, Hahne et al. demonstrated that CSP feature showed a higher robustness against noise than time domain (TD) feature for myoelectric control [17]. However, they did not investigate the performance of CSP feature in the presence of electrode shift. Huang et al. also used an improved CSP for EMG classification, but they targeted LD EMG and did not consider the problem of electrode shift [24].

In this study, we investigate whether the CSP of HD EMG signals can improve the myoelectric control performance under electrode shift for eleven classes of hand and wrist motions. We test nine able-bodied subjects. The performance of CSP feature is compared with the commonly used TD, TDAR and Variog features. Linear discriminant analysis (LDA) classifiers are used to process the EMG data.

## Methods

### Subjects

Nine able-bodied subjects (eight males and one female; aged 22–27; referenced as Sub1-Sub9) participated in the experiment. The subjects had no neurological disorders. This work was approved by the Ethics Committee of Shanghai Jiao Tong University. All subjects participating in the experiment signed informed consent and the procedures were in compliance with the Declaration of Helsinki.

### Experiment setup

Eleven classes of hand and wrist motions were performed by the subjects in order, i.e., hand close (HC), hand open (HO), key grip (KG), tip prehension (TP), wrist flexion (WF), wrist extension (WE), radial deviation (RD), ulnar deviation (UD), forearm supination (FS), forearm pronation (FP) and “no movement” (NM). In each trial, the subjects were asked to perform each motion for 10 s. Ten trials were performed by each subject. To avoid fatigue, the subjects had a 1-min rest between each trial.

### Data acquisition

**Fig. 1:**
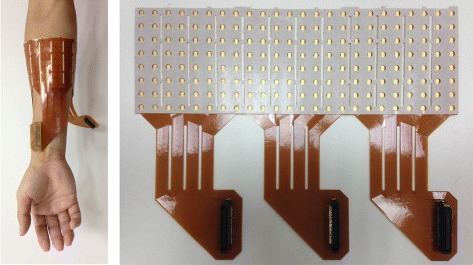
Position of the HD EMG grid and the HD EMG grid of 192 electrodes used in the experiments

Monopolar surface EMG signals were measured and collected using a grid of 192 electrodes (3 semi-disposable adhesive matrix, 64 electrodes, ELSCH064NM3) composed by 8 rows and 24 columns, with 10 mm interelectrode distance (IED) (Fig. 1). The skin surface of forearm was rubbed lightly with alcohol to reduce impedance. The grid was mounted around the circumference of the forearm (Fig. 1), starting from the ulnar bone. The grid was mounted on the skin by adhesive foam and a reference electrode was mounted at the wrist. The matrixes were connected to a multichannel surface EMG amplifier (EMG-USB2 +, OT Bioelettronica, Torino, Italy) and the signals were amplified with a gain of 500, band-pass filtered (pass band 10–500 Hz), sampled at 2048 Hz, and A ∖D converted with 12-bit resolution.

### Common spatial patterns

CSP is a supervised two-class method to design linear spatial filters simultaneously maximizing the variance of one class and minimizing the variance of another class [22]. In this way, the classes can be maximally separated by their variances. CSP is widely used in motor imaginary-based brain computer interface (BCI) for classification of EEG signals [23, 25].

The raw EMG signals of class *j* and class *k* were represented as *X*_*j*_ and *X*_*k*_ with dimensions *c*×*l*, where *c* was the number of channels, and *l* was the number of samples per each channel (here *l* was 408). The objective was to find the *ω* of the spatial filter *y*=*ω*^*T*^*X*, which maximized the variance of class *j* and minimized the variance of class *k*. Thus, the optimization process was formulated as following:

(1) $$ \omega = \mathop{\arg\max}\limits_{\omega} \frac{{{\omega^{T}}{\sum_{j}}\omega}}{{{\omega^{T}}{\sum_{k}}\omega }}  $$

where ${\sum _{j}} = 1/(n - 1)*{X_{j}}*{X_{j}}^{T}$ and ${\sum _{k}} = 1/(n - 1)*{X_{k}}*{X_{k}}^{T}$ were the covariance matrix of class *j* and class *k* respectively.

This was realized by finding the matrix *W* that simultaneously diagonalized both ${\sum _{j}}$ and ${\sum _{k}}$:

(2) $$ W{{\sum\nolimits}_{j}} {{W^{T}}} = {D_{j}},  $$

(3) $$ W{\sum\nolimits}_{k} {{W^{T}}} = {D_{k}},  $$

(4) $$ {D_{j}} + {D_{k}} = I.  $$

The row vectors of *W* were *c* spatial filters. Applying the full filter matrix *W* to the raw EMG signals would give *c* output signals *Y*=*W*∗*X*, which were called components. The variance of each component for class *j* was indicated by the corresponding eigenvalue of *D*_*j*_, for class *k* of *D*_*k*_. With the constraint (4), the eigenvector corresponding to the largest eigenvalue for *D*_*j*_ would had the smallest eigenvalues for *D*_*k*_, and the eigenvector corresponding to the largest eigenvalue for *D*_*k*_ would had the smallest eigenvalues for *D*_*j*_. These two eigenvectors were chosen as the spatial filters in this study.

### Multiclass CSP

Since there were eleven motion classes in this study, we extended the two-class CSP into multiclass CSP by using one versus one (CSP-OvO) and one versus rest (CSP-OvR) scheme [17].

In the CSP-OvO scheme, the two-class CSP was designed for all possible class combinations. The filters were chosen in the same way as in the two-class CSP. Thus, there were *M*=*N*∗(*N*−1)/2 combinations for *N* classes. The features of all selected components were concatenated into one feature vector.

In the CSP-OvR scheme, each filter was designed to maximize the variance of one class and minimize the average of the variances of all other classes. The filters were chosen in the same way as in the two-class CSP. This process was repeated for all classes. Thus, there was *N* combinations for *N* classes. The features of all selected components were concatenated into one feature vector.

### Feature extraction

The logarithm of the variances of the selected CSP components were calculated as features in the CSP-OvO and CSP-OvR scheme. Here, the length of analysis window was set to 200 ms and the increment of two adjacent windows was set to 50 ms. The length and the increment were chosen to ensure response time of the system was below 300 ms for reducing users’ perceived lag [5]. A feature set was computed on each of the CSP component, and then concatenated to form a feature vector.

To compare the proposed feature extraction method with the state of the art technology, TD features, TDAR features and Variog features, which were effective and robust with electrode shift [2, 5, 11, 18, 26, 27], were used in this study. These features were extracted using the same window length and the same increment as those specified in above paragraph.

### Classification

As a simple and efficient classifier, the LDA classifier has been widely used for pattern recognition of EMG signals [7, 28]. Researchers have presented in previous studies that the LDA classifier can have the comparable performance to other more sophisticated classifiers [29] and generalizes better than the nonlinear multilayer perceptron classifier with electrode shift [11]. Hence, the LDA classifier was employed to identify the CSP features (CSP-OvO and CSP-OvR) and the two classic features (TD and TDAR) in this study. Since the Variog features performed better with support vector machine (SVM) classifier compared with LDA classifier [18], the SVM classifier was employed to identify the Variog features in this study [30]. A five-fold cross-validation procedure was used. Four fifths of the data were randomly selected and used as a training set to train the LDA classifier, while the remaining one fifth were used as a testing set.

### Electrodes shift

**Fig. 2:**
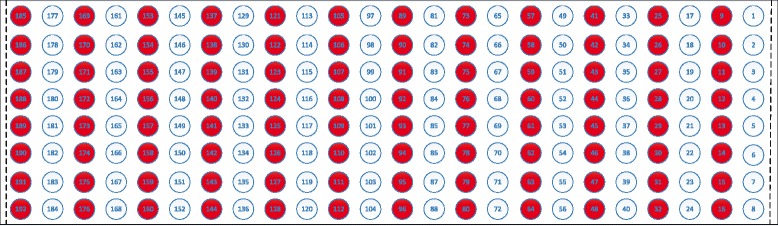
Shift transversal to the direction of the muscle fibers. Shift leftwards (ST1): the white color electrodes were used for training, while the red color electrodes were used for testing. Shift rightwards (ST2): the red color electrodes were used for training, while the white color electrodes were used for testing

**Fig. 3:**
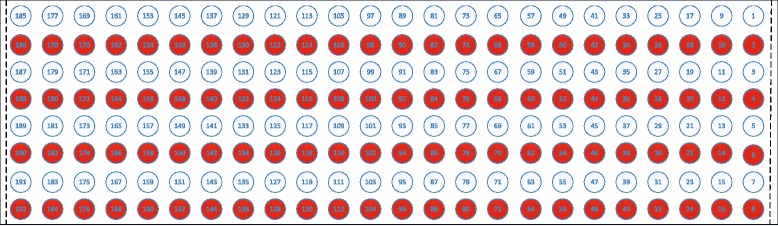
Shift longitudinal to the direction of the muscle fibers. Shift downwards (SL1): the white color electrodes were used for training, while the red color electrodes were used for testing. Shift upwards (SL2): the red color electrodes were used for training, while the white color electrodes were used for testing

Shift transversal and longitudinal to the direction of the muscle fibers were taken into consideration. We expected that shift in longitudinal or transversal direction would be the extreme situation. Meanwhile, the influence of electrode shift occurring along both axes would be between the influences of electrode shift in longitudinal and transversal directions. Since a shift of 10 mm or less was considered more likely in clinical applications [11], the shift distance was chosen as 10 mm to simulate the worst shift situation in the current study. To simulate the shift transversal to the direction of the muscle fibers, half of the columns were used for training and the remaining half for testing, which corresponded to a 10-mm shift for a configuration of 96 electrodes. Figure 2 shows the shift in transversal direction of the muscle fibers. Shift leftwards (ST1): the white color electrodes were used for training, while the red color electrodes were used for testing. Shift rightwards (ST2): the red color electrodes were used for training, while the white color electrodes were used for testing. To provide a control for transversal direction shift, the same color electrodes in Fig. 2 were used for both training and testing, referred as ST. It should be noted that the electrodes distance in the transversal and longitudinal direction was 20 mm and 10 mm respectively. Similar method was used to simulate the shift in longitudinal direction of the muscle fibers (Fig. 3). To provide a control for longitudinal direction shift, the same color electrodes in Fig. 3 were used for both training and testing, referred as SL. It should be noted that the electrodes distance in the transversal and longitudinal direction was 10 mm and 20 mm respectively.

### Quantification of feature space

To investigate the variations in the EMG feature space before and after the electrode shift, relative center shift (RCS) was defined in the current study. RCS was defined as the ratio between the mean value of the Mahalanobis distance of the same motion before and after the electrode shift across *N* motions and the mean value of the Mahalanobis distance of the different motions across *N* motions after the electrode shift:

(5) $$ {}RCS = \frac{(N - 1)\sum\limits_{i = 1}^{N} {\sqrt {{{({\mu_{i}} - {\mu_{si}})}^{T}}{{\left(\frac{{{S_{i}} + {S_{si}}}}{2}\right)}^{- 1}}({\mu_{i}} - {\mu_{si}})}}}{{\sum\limits_{i = 1}^{N} {\sum\limits_{j = 1,j \ne i}^{N} {\sqrt {{{({\mu_{sj}} - {\mu_{si}})}^{T}}{{\left(\frac{{{S_{sj}} + {S_{si}}}}{2}\right)}^{- 1}}({\mu_{sj}} - {\mu_{si}})}} } }}  $$

where *μ*_*i*_ and *μ*_*si*_ were the centroid of the ellipsoid of motion *i* before and after the electrode shift, *S*_*i*_ and *S*_*si*_ were the covariance of the data for motion *i* before and after the electrode shift.

The value of RCS was positively correlated to the relative center shift in the EMG feature space.

As different feature sets would have different dimensionality of feature vector, prior to computation of RCS, the Fisher linear discriminant (FLD) [31] was adopted to reduce the dimension of feature vectors to the same level of *N*−1, where *N* is the number of motions, which was eleven here. Since the Variog features were identified by SVM classifier but not LDA classifier, the FLD was not suitable to process the Variog features. Therefore, the RCS was not computed on the Variog features.

### Visualization of CSP patterns

**Fig. 4:**
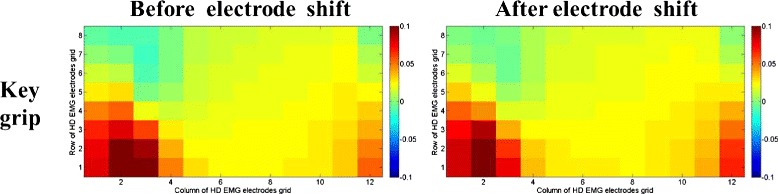
Last CSP pattern of motion 1 and motion 4 for CSP-OvO extension scheme in transversal direction shift (ST1). Left and right columns were the CSP patterns before and after electrode shift

**Fig. 5:**
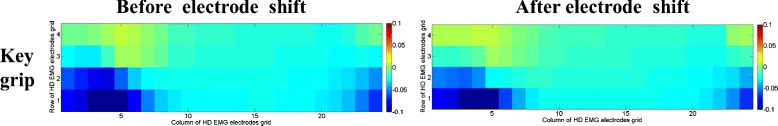
Last CSP pattern of motion 1 and motion 4 for CSP-OvO extension scheme in longitudinal direction shift (SL1). Left and right columns were the CSP patterns before and after electrode shift

**Fig. 6:**
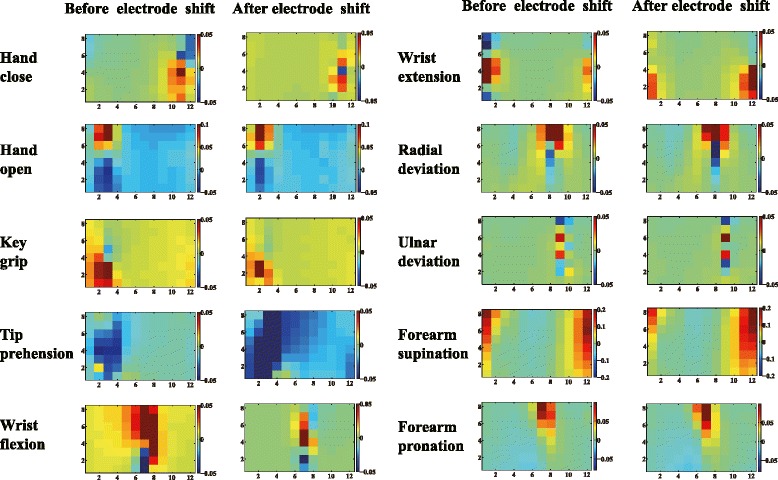
First CSP pattern of each active motion and rest motions for CSP-OvR extension scheme in transversal direction shift (ST1). First and second columns were the CSP patterns of the first five active motions (HC, HO, KG, TP and WF) before and after electrode shift. Third and fourth columns were the CSP patterns of the last five active motions (WE, RD, UD, FS and FP) before and after electrode shift

**Fig. 7:**
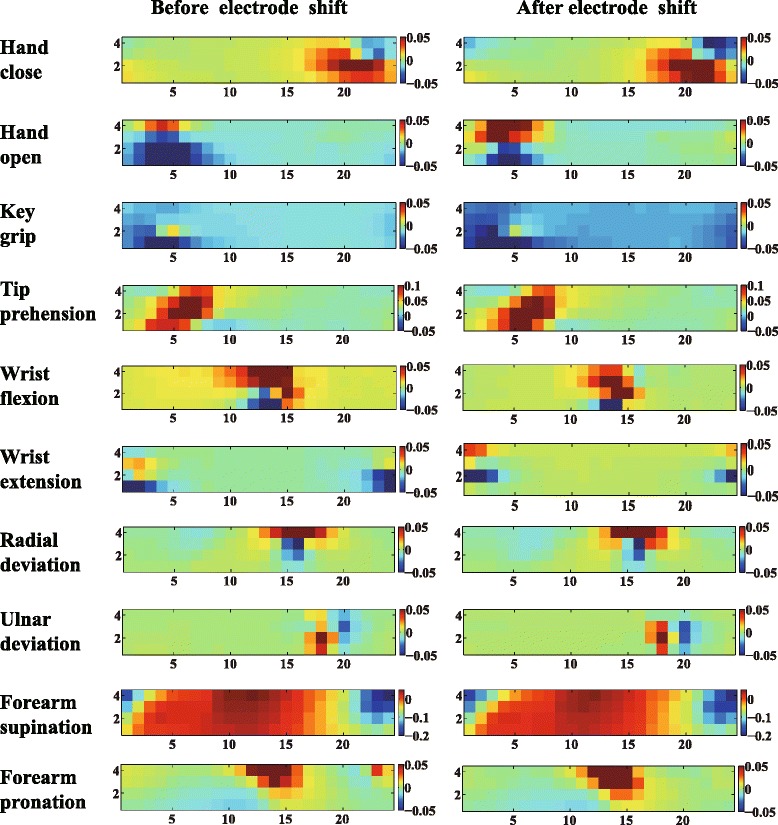
First CSP pattern of each active motion and rest motions for CSP-OvR extension scheme in longitudinal direction shift (SL1). Left and right columns were the CSP patterns before and after electrode shift

To understand the improvements of the CSP features, the corresponding patterns of the motions before and after the electrode shift were visualized for a representative subject (Sub3). CSP patterns were columns of the inverse of filter matrix *W*. The *i*th pattern represented the source signal distribution to the sensors that produced activity in the *i*th CSP component. CSP patterns provided valuable information about the underlying electrophysiology processes and the related muscles. Contrary to the EMG amplitude patterns, which only showed muscle activation information, the CSP patterns emphasized the locations that provided most information to discriminate different motions. Figures 4 and 5 show the last CSP patterns of motion 1 and motion 4 for CSP-OvO extension scheme in the transversal direction shift (ST1) and longitudinal direction shift (SL1) respectively. Figures 6 and 7 show the first CSP pattern of each active motion and rest motions for CSP-OvR extension scheme in the transversal direction shift (ST1) and longitudinal direction shift (SL1) respectively.

### Statistical analysis

A two-way repeated measures ANOVA was used to analyze CA. The ANOVA included the following two factors: Shift (ST1, ST2, SL1 and SL2) and Feature (CSP-OvO, CSP-OvR, TD, TDAR and Variog). Similarly, a two-way repeated measures ANOVA was used to analyze RCS. The ANOVA included the following two factors: Shift (ST1, ST2, SL1 and SL2) and Feature (CSP-OvO, CSP-OvR, TD and TDAR). In all ANOVA tests, the full model was conducted first. When a significant interaction was detected, a simple-effects analysis was conducted by fixing the levels of one of the interacting factors. When no interaction was detected, a reduced ANOVA model with only the main factors was performed. Whenever significance was detected for the main factors, a Tukey comparison was performed. Only a significant difference was reported for these comparison tests. The significance level for all tests was *p*<0.05.

## Results

### Classification accuracy

**Fig. 8:**
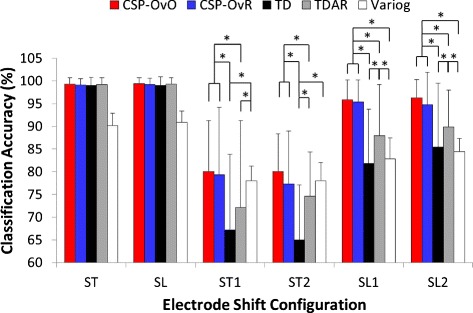
Average CA across all subjects for five features: CSP-OvO, CSP-OvR, TD, TDAR and Variog. Error bars represented the standard deviation. The tests were marked by * in which significance were found between different features

Figure 8 shows the average CA of all features (CSP-OvO, CSP-OvR, TD, TDAR and Variog) across all subjects for the half grid configuration of 96 electrodes (ST and SL) and the different shift configurations (ST1, ST2, SL1 and SL2). The average CA of CSP-OvO and CSP-OvR was slightly higher than that of TD and was comparable with that of TDAR for the half grid configuration without electrode shift (ST and SL). The average CA of CSP-OvO, CSP-OvR, TD and TDAR was 8 % higher than that of Variog for the half grid configuration without electrode shift (ST and SL). Since the average CA of all features without electrode shift was over 90 %, it demonstrated that the half grid configuration without electrode shift was sufficient to provide good myoelectric control performance for all features (CSP-OvO, CSP-OvR, TD, TDAR and Variog). However, the average CA for TD was decreased to 67.2 % in ST1, 65.0 % in ST2, 81.9 % in SL1, and 85.4 % in SL2; the average CA for TDAR was decreased to 72.1 % in ST1, 74.5 % in ST2, 87.9 % in SL1, and 89.9 % in SL2; the average CA for Variog was decreased to 78 % in ST1, 78 % in ST2, 82.8 % in SL1, and 84.4 % in SL2. The average CA for CSP features (CSP-OvO and CSP-OvR) was ∼80 % in the electrode shift in transversal direction (ST1 and ST2) and ∼95 % in the electrode shift in longitudinal direction (SL1 and SL2) respectively. Thus, the CSP features (CSP-OvO and CSP-OvR) were more robust than the commonly used features (TD, TDAR and Variog) in the presence of electrode shift.

The two-way ANOVA revealed a statistically significant interaction between Shift and Feature (*p*<0.001). The simple-effects analysis was conducted to break down the ANOVA into subsequent one-way ANOVA, looking separately at the ST1, ST2, SL1 and SL2 for main effect of Feature.

For the Shift ST1, the one-way ANOVA revealed a main effect of Feature (*p*<0.001). Tukey comparison showed that the CA of CSP-OvO was not significantly different with that of CSP-OvR (*p*=0.997) and Variog (*p*=0.869) but significantly higher than that of TD (*p*<0.001) and TDAR (*p*=0.002); the CA of CSP-OvR was not significantly different with that of Variog (*p*=0.973) but significantly higher than that of TD (*p*<0.001) and TDAR (*p*=0.006); the CA of TD was not significantly different with that of TDAR (*p*=0.138) but significantly lower than that of Variog (*p*<0.001); the CA of TDAR was significantly lower than that of Variog (*p*=0.04).

For the Shift ST2, the one-way ANOVA revealed a main effect of Feature (*p*<0.001). Tukey comparison showed that the CA of CSP-OvO was not significantly different with that of CSP-OvR (*p*=0.531) and Variog (*p*=0.777) but significantly higher than that of TD (*p*<0.001) and TDAR (*p*=0.019); the CA of CSP-OvR was not significantly different with that of TDAR (*p*=0.536) and Variog (*p*=0.995) but significantly higher than that of TD (*p*<0.001) and TDAR (*p*=0.006); the CA of TD was significantly lower than that of TDAR (*p*<0.001) and Variog (*p*<0.001); the CA of TDAR was not significantly different with that of Variog (*p*=0.3).

For the Shift SL1, the one-way ANOVA revealed a main effect of Feature (*p*<0.001). Tukey comparison showed that the CA of CSP-OvO was not significantly different with that of CSP-OvR (*p*=0.995) but significantly higher than that of TD (*p*<0.001), TDAR (*p*<0.001) and Variog (*p*<0.001); the CA of CSP-OvR was significantly higher than that of TD (*p*<0.001), TDAR (*p*<0.001) and Variog (*p*<0.001); the CA of TD was not significantly different with that of Variog (*p*=0.944) but significantly lower than that of TDAR (*p*<0.001); the CA of TDAR was significantly higher than that of Variog (*p*<0.001).

For the Shift SL2, the one-way ANOVA revealed a main effect of Feature (*p*<0.001). Tukey comparison showed that the CA of CSP-OvO was not significantly different with that of CSP-OvR (*p*=0.783) but significantly higher than that of TD (*p*<0.001), TDAR (*p*<0.001) and Variog (*p*<0.001); the CA of CSP-OvR was significantly higher than that of TD (*p*<0.001), TDAR (*p*=0.002) and Variog (*p*<0.001); the CA of TD was not significantly different with that of Variog (*p*=0.933) but significantly lower than that of TDAR (*p*=0.006); the CA of TDAR was significantly higher than that of Variog (*p*<0.001).

**Fig. 9:**
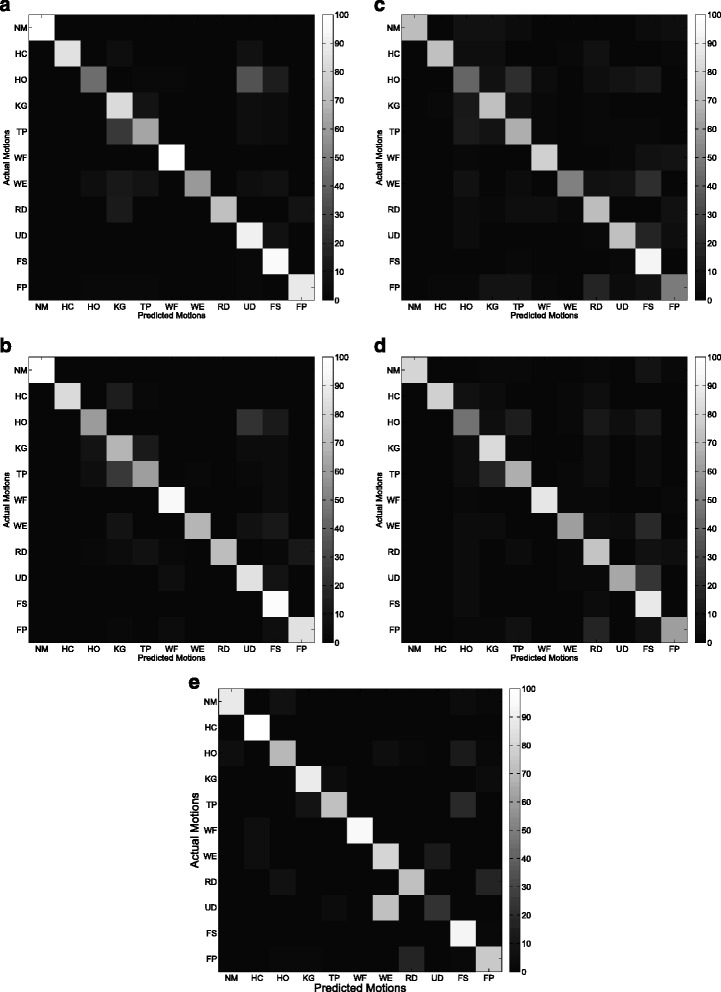
Average confusion matrix of the five features across all subjects in ST1. **a** CSP-OvO. **b** CSP-OvR. **c** TD. **d** TDAR. **e** Variog

**Fig. 10:**
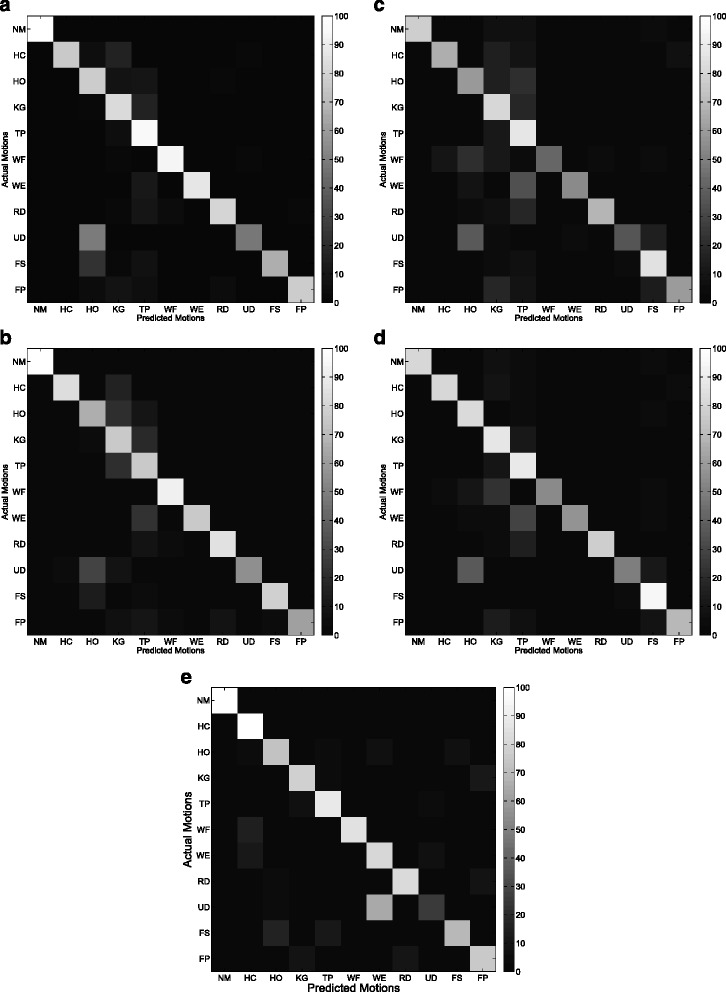
Average confusion matrix of the five features across all subjects in ST2. **a** CSP-OvO. **b** CSP-OvR. **c** TD. **d** TDAR. **e** Variog

**Fig. 11:**
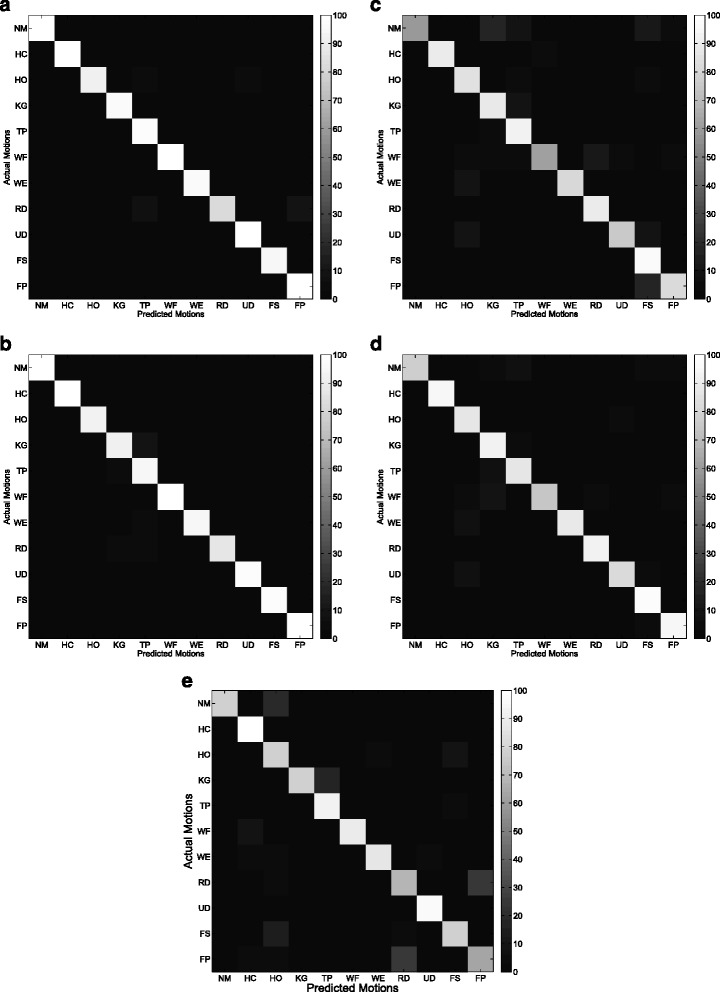
Average confusion matrix of the five features across all subjects in SL1. **a** CSP-OvO. **b** CSP-OvR. **c** TD. **d** TDAR. **e** Variog

**Fig. 12:**
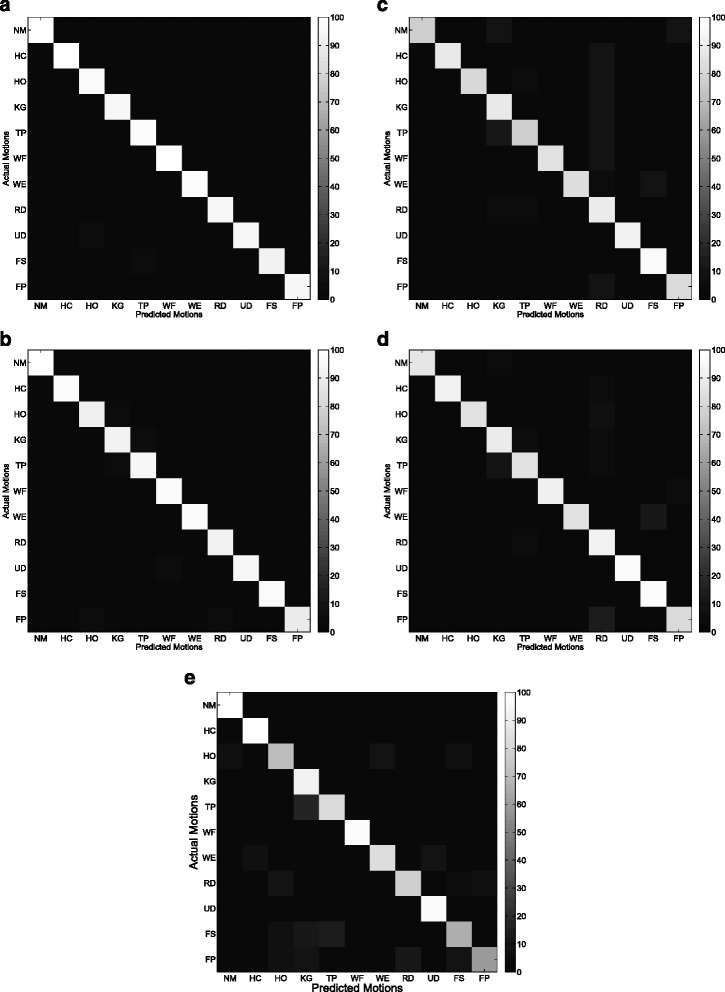
Average confusion matrix of the five features across all subjects in SL2. **a** CSP-OvO. **b** CSP-OvR. **c** TD. **d** TDAR. **e** Variog

Figures 9 and 10 show the average confusion matrix of the five features (CSP-OvO, CSP-OvR, TD, TDAR and Variog) across all subjects in ST1 and ST2 respectively. We could find that the improvements of CSP features were mainly from NM, WF and UD in ST1 and were mainly from NM, WF and WE in ST2. Figures 11 and 12 show the average confusion matrix of the five features across all subjects in SL1 and SL2 respectively. We could find that the improvements of CSP features were mainly from NM, WF and UD in SL1 and were mainly from NM, TP, and WE in SL2. Comparing (a) and (b) of Figs 9, 10, 11 and 12, we found that the CA for each motion was similar between the two CSP features in all shift configurations (ST1, ST2, SL1 and SL2). Furthermore, we found the misclassifications of one motion vs. another (e.g. HO vs. UD) were also similar between the two CSP features in all shift configurations. These results demonstrated that the separability from one motion to another was very similar between the two CSP features and could explain why the classification performance of the two CSP features was not significant different in all shift configurations. Comparing (a)–(d) and (e) of Figs. 9, 10, 11 and 12, we found that the misclassifications of one motion vs. another of Variog feature were pretty different from that of the other four features. We suggested that this phenomenon was induced by the different type of classifier that the Variog feature used.

### Shift in EMG feature space

**Fig. 13:**
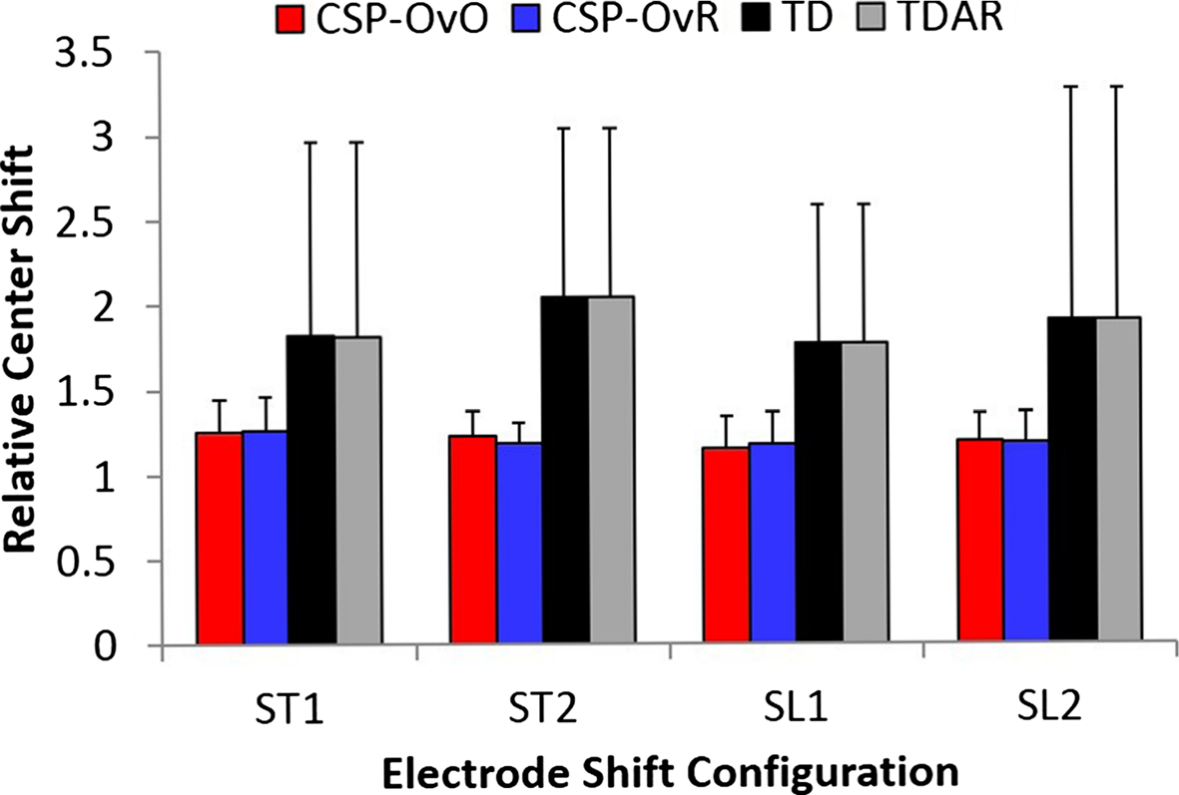
Average RCS across all subjects for four features: CSP-OvO, CSP-OvR, TD, and TDAR. Error bars represented the standard deviation

Figure 13 shows the average RCS of the four features (CSP-OvO, CSP-OvR, TD and TDAR) across all subjects in the different shift configurations (ST1, ST2, SL1 and SL2). The average RCS of CSP-OvO changed from 1.15 to 1.25. The average RCS of CSP-OvR changed from 1.18 to 1.27. The average RCS of TD changed from 1.77 to 2.04. The average RCS of TDAR changed from 1.77 to 2.04. The average RCS of CSP features (CSP-OvO and CSP-OvR) was about two thirds of that of classic features (TD and TDAR) in all shift configurations (ST1, ST2, SL1 and SL2).

The two-way ANOVA revealed a statistically significant main effect of Feature (*p*<0.001). No other significant two-way interaction or main effect was revealed. For the factor of Feature, Tukey comparison showed that the RCS of CSP-OvO was not significantly different with that of CSP-OvR (*p*=1.0), but significant smaller than that of TD (*p*<0.001) and TDAR (*p*<0.001). It also showed that the RCS of CSP-OvR was significant smaller than that of TD (*p*<0.001) and TDAR (*p*<0.001). However, the RCS of TD was not significantly different with that of TDAR (*p*=1.0). The results demonstrated that the significant improvement in CA of the CSP features (CSP-OvO and CSP-OvR) was induced by the significantly smaller RCS in the feature space compared with the classic features (TD and TDAR). Unlike the CSP features (CSP-OvO and CSP-OvR), the significant difference in CA between TDAR and TD was not reflected on the RCS. The main reason was likely that the difference in CA between TDAR and TD was much smaller compared with the difference in CA between the CSP features and the classic features.

### Interpretation of improvement in patterns of CSP features

Figures 4 and 5 show the last CSP patterns of motion 1 and motion 4 for CSP-OvO extension scheme in the transversal direction shift (ST1) and longitudinal direction shift (SL1) respectively. Figures 6 and 7 show the first CSP pattern of each active motion and rest motions for CSP-OvR extension scheme in the transversal direction shift (ST1) and longitudinal direction shift (SL1) respectively. We found that the locations emphasized by the CSP patterns before and after the shift were very similar. We believed that this was due to the underlying electrophysiology processes were not changed even in the presence of electrode shift. Thus, the CSP patterns of the EMG signals before electrode shift could emphasize the most discriminative locations after electrode shift and improve the CA in all electrode shift configurations (ST1, ST2, SL1 and SL2).

## Discussion

As shown in Fig. 8, CSP features (CSP-OvO and CSP-OvR) significantly improved the CA over 10 % with respect to TD features in all shift configurations (ST1, ST2, SL1 and SL2) (*p*<0.05). The CSP-OvO feature achieved the highest average CA in all electrode configurations (ST, SL, ST1, ST2, SL1 and SL2) and significantly improved the average CA over 5 % with respect to TDAR features in all shift configurations (*p*<0.05). Except shift configuration ST2, the CSP-OvR feature significantly improved the average CA with respect to TDAR features in shift configurations ST1, SL1 and SL2 (*p*<0.05). Except transversal shift configurations (ST1 and ST2), the CSP features (CSP-OvO and CSP-OvR) significantly improved the average CA with respect to Variog features in longitudinal shift configurations (SL1 and SL2) (*p*<0.05). The CSP features (CSP-OvO and CSP-OvR) could achieve the average CA of ∼80 % in transversal direction shift (ST1 and ST2) and ∼95 % in longitudinal direction shift (SL1 and SL2). Thus, the CSP features could improve robustness against electrode shift for myoelectric control with respect to classic features.

Although there was no significant difference between the CA of CSP-OvO feature and that of CSP-OvR feature in all electrode configurations in Fig. 8, the average CA of CSP-OvO feature was slightly higher than that of CSP-OvR feature in all electrode configurations. We attributed this to the fact that the number of features extracted in CSP-OvO scheme was much more than the number of features extracted in CSP-OvR scheme. Therefore, the CSP-OvO feature extracted more helpful information for classification from the HD EMG signals with respect to the CSP-OvR feature. Furthermore, the results showed that the CSP features (CSP-OvO and CSP-OvR) performed best in longitudinal shift configurations (SL1 and SL2). We believed that this was presumably due to the fact that the electrode configuration was shifted in this case along the muscle fiber direction.

The results also showed that TDAR features significantly improved the CA with respect to TD features in shift configurations ST2, SL1 and SL2 (*p*<0.05). These confirmed the result that TDAR features significantly reduced sensitivity to electrode shift compared with TD features of a previous study [11]. Furthermore, the results showed that the Variog features significantly improved the average CA with respect to TD features in shift configurations ST1 and ST2 (*p*<0.05) and improved the average CA with respect to TDAR features in shift configuration ST1. However, the CA of the Variog features was not significantly different with that of TDAR features in shift configuration ST2 (*p*=0.3) and significantly lower than that of TDAR features in longitudinal direction shift (SL1 and SL2) (*p*<0.05). These results were partially consistent with the results of previous study [18]. Since parameters choosing was very important when using the Variog features and SVM classifiers, we suggested this might be due to that we could not find the optimized parameters in the current study. Moreover, this might be due to the number of motions considered in the current study was eleven which was larger than seven in that previous study.

As shown in Fig. 13, the average RCS of CSP features (CSP-OvO and CSP-OvR) across all subjects was significantly smaller than that of classic features (TD and TDAR) in all shift configurations (*p*<0.001). Since the average value of the feature vector of each motion and the covariance of all EMG data determined the parameters of the LDA classifier, the smaller RCS indicated that the LDA classifier trained before the electrode shift was more suitable for identifying the EMG data after the electrode shift. Thus, the CA of the features with smaller RCS should be greater than that with larger RCS. Here, we attributed that the improvement of CSP features (CSP-OvO and CSP-OvR) with respect to classic features (TD and TDAR) was induced by the relatively smaller RCS compared with the classic features.

For noise investigations, Hahne et al. have evaluated the performance of CSP features with a high baseline noise of individual channels and proved that the CSP features outperformed the classic features [17]. Thus, we did not test this effect, but only concentrated on the electrode shift in the current study.

The results showed that the proposed CSP features could improve the robustness against electrode shift for myoelectric control compared with the commonly used features. However, a limitation existed in the current study was that the proposed CSP features were not suitable for LD EMG. Geng et al. used CSP method to select LD channels from HD EMG, but they targeted channel selection and did not consider the problem of electrode shift [19]. Huang et al. also used an improved CSP for EMG classification, but they targeted LD EMG and did not consider the problem of electrode shift [24]. For LD EMG application, the proposed CSP features should be modified to common spatio-spectral pattern (CSSP) features and then evaluate their performance against electrode shift. In CSSP, several finite impulse response (FIR) spectral filters were embedded into CSP to constitute a spatio-spectral filter [20]. Since the embedded FIR filters would improve the number of channels for CSP, it could make the CSP suitable for LD EMG. We will investigate the performance against electrode shift of CSSP features for LD EMG application in the future.

As this work is an off-line analysis, an on-line study should be taken into account. In the future, the CSP features (CSP-OvO and CSP-OvR) will be tested in real-time experiments measured by three performance metrics, i.e. motion completion rate, motion completion time and motion selection time [32, 33]. There is a limitation in the current study that the subjects are intact-limb subjects. Although Scheme et al. showed that the results from intact-limb subjects could be generalized to amputees [34], the CSP features (CSP-OvO and CSP-OvR) should be tested on amputees in future work. To test the applicability of CSP features in practice, whether the computation capability of current micro-controller is enough for the analysis of HD EMG signals in myoelectric control should be investigated in the future.

## Conclusion

This study evaluated whether the CSP of HD EMG signals could improve the myoelectric control performance under electrode shift for eleven classes of hand and wrist motions. Compared with the TD features, the CSP features significantly improved the CA over 10 % in all shift configurations (ST1, ST2, SL1 and SL2). Compared with the TDAR features, a. the CSP-OvO feature significantly improved the average CA over 5 % in all shift configurations; b. the CSP-OvR feature significantly improved the average CA in shift configurations ST1, SL1 and SL2. Compared with the Variog features, the CSP features significantly improved the average CA in longitudinal shift configurations (SL1 and SL2). It demonstrated that CSP of HD EMG signals could improve robustness against electrode shift for myoelectric control with respect to the commonly used features.
